# Toxicological and efficacy assessment of post-transition metal (Indium) phthalocyanine for photodynamic therapy in neuroblastoma

**DOI:** 10.18632/oncotarget.11942

**Published:** 2016-09-10

**Authors:** Monica Neagu, Carolina Constantin, Mircea Tampa, Clara Matei, Andreea Lupu, Emilia Manole, Rodica-Mariana Ion, Concettina Fenga, Aristidis M. Tsatsakis

**Affiliations:** ^1^ Faculty of Biology, University of Bucharest, Romania; ^2^ Immunobiology Laboratory and Alternative Testing Multi-Disciplinary Team, “Victor Babeş” National Institute of Pathology, Bucharest, Romania; ^3^ Dermatology Department, “Carol Davila” University of Medicine and Pharmacy, Bucharest, Romania; ^4^ Research Center, Colentina Clinical Hospital, Bucharest, Romania; ^5^ Nanomedicine Research Group, National Institute of R&D for Chemistry and Petrochemistry – ICECHIM, Bucharest, Romania; ^6^ Materials Engineering Department, Valahia University of Targovişte, Romania; ^7^ Section of Occupational Medicine, University of Messina, Messina, Italy; ^8^ Department of Toxicology and Forensic Sciences, Faculty of Medicine, University of Crete, Heraklion, Greece

**Keywords:** photodynamic therapy, neuroblastoma, indium-phthalocyanine, toxicology, cellular impedance

## Abstract

Metallo-phthalocyanines due to their photophysical characteristics as high yield of triplet state and long lifetimes, appear to be good candidates for photodynamic therapy (PDT). Complexes with diamagnetic metals such as Zn^2+^, Al^3+^ Ga^3+^ and In^3+^meet such requirements and are recognized as potential PDT agents. Clinically, Photofrin^®^ PDT in neuroblastoma therapy proved in pediatric subjects diagnosed with progressive/recurrent malignant brain tumors increased progression free survival and overall survival outcome. Our study focuses on the dark toxicity testing of a Chloro-Indium-phthalocyanine photosensitizer (In-Pc) upon SH-SY5Y neuroblastoma cell line and its experimental *in vitro* PDT. Upon testing, In-Pc has shown a relatively high singlet oxygen quantum yield within the cells subjected to PDT (0.553), and 50 μg/mL IC50. Classical toxicological and efficacy assessment were completed with dynamic cellular impedance measurement methodology. Using this technology we have shown that long time incubation of neuroblastoma cell lines in In-Pc (over 5 days) does not significantly hinder cell proliferation when concentration are ≤ 10 μg/mL. When irradiating neuroblastoma cells loaded with non-toxic concentration of In-Pc, 50% of cells entered apoptosis. Transmission electron microscopy has confirmed apoptotic characteristics of cells. Investigating the proliferative capacity of the *in vitro* treated cells we have shown that cells that “escape” the irradiation protocol, present a reduced proliferative capacity. In conclusion, In-Pc represents another photosensitizer that can display sound PDT properties enhancing neuroblastoma therapy armentarium.

## INTRODUCTION

Metal-based phthalocyanines are currently used in industrial applications, but these compounds have also properties that make them prospective photosensitizers. Compounds with photosensitizing properties, produce reactive oxygen species upon exposure to light, inducing apoptosis/necrosis pathways and having a long time history in photodynamic therapy (PDT)[1, 2]. In cancer, PDT is already approved in several pathologies and the race of designing new and more efficient photosensitizers (PS) is on. PDT implies the use of three mandatory elements: light, oxygen and a photosensitizer [3]. Hence PDT consists in application of the PS, whether systemic or topical, and after a time interval, in low light, PS accumulates in the tumor tissue. Subsequently, PS's accumulated molecules will be activated upon irradiation with a particular wavelength laser light inducing photochemical and photophysical processes. These processes reside in the energy transfer to the nearby oxygen, transfer that generates reactive oxygen species (mainly singlet oxygen, hydroxyl radical, and superoxide anions). As a consequence an oxidative stress induced by PDT is generated, cellular organelles and membranes become damaged, a process known as tumor photodamage. Moreover, singlet oxygen induces also blocking of the blood supply to the tumor, by microvascular acute injury and blood vessel blockage [4–8]. Phthalocyanines as PSs have the ability to use a highly conjugated delocalized π-electrons system and, having an increased structural flexibility, enable them to accommodate different substitution groups in the peripheral position of the Pc ring or to be the subject of different transition metals insertion. Metallo-phthalocyanines (MePcs) due to their photophysical characteristics have high yield of triplet state and long lifetimes. Complexes with diamagnetic metals such as Zn^2+^, Al^3+^ and Ga^3+^ meet such requirements and are recognized as such [9, 10]. The insertion of heavy metals enhances the inter-system crossing rates. This is important because triplet lifetimes are significantly longer than singlet lifetimes, and the triplet-triplet transition has an enhanced absorption compared to corresponding singlet-singlet transition [11].

In comparison with aluminium derivatives, the chemistry of indium phthalocyanines is less investigated. Introducing heavy metal in the complex provides increased quantum yields of triplet state and singlet oxygen, increasing hence its photodynamic properties [12].

Choosing indium as a central metal is important because in its (bio)compounds, indium holds the oxidation state In^3+^ which allows an axially chemical substitution forming complexes that are very promising for future PDT applications [13]. Chloride Indium (III) phthalocyanine derivatives (In-Pc) having chlorine atoms at axial positions and bulky peripheral substituents have a low aggregation capacity in solution. All these taken together make In-Pc worth developing as PDT agent.

We have chosen this *in vitro* tumor model based on several clinical reasons. Therapy in this type of tumor is limited, literature focusing on neuroblastoma (NB) and PDT is scarce as in the last 20 years only a dozen of studies have been published.

NB is frequent in childhood and infancy, being the most common extracranial solid cancer [14]. A therapeutical protocol with Photofrin^®^ and light energy has registered in pediatric subjects diagnosed with progressive/recurrent malignant brain tumors an increased free survival and overall survival [14].The age-standardized incidence rate (ASR) of NB in Europe steadily increases and this tendency owes to the incidence in infants were 52.6 cases *per* million children are reported. The reported overall 5-year survival is 59%, although several important steps were taken in therapy [15]. NB is a very heterogeneous disease spanning a wide range of clinical evolution, from low-risk disease with good outcome to difficult to treat high-risk disease even when multi-modal therapies are approached [16, 17, 18, 19].

All these arguments come in favor of studying the possibility to use phthalocyanine's class of photosensitizers and to test them in neuroblastoma cell lines. Accordingly, our study focuses on the dark toxicity testing of a In-Pc photosensitizer upon SH-SY5Y cell line and its effect in experimental *in vitro* photodynamic therapy approach in order to establish the safety toxicological domain and its anti-tumoral efficacy.

## RESULTS

### Physical and chemical characterization

**Figure 1:**
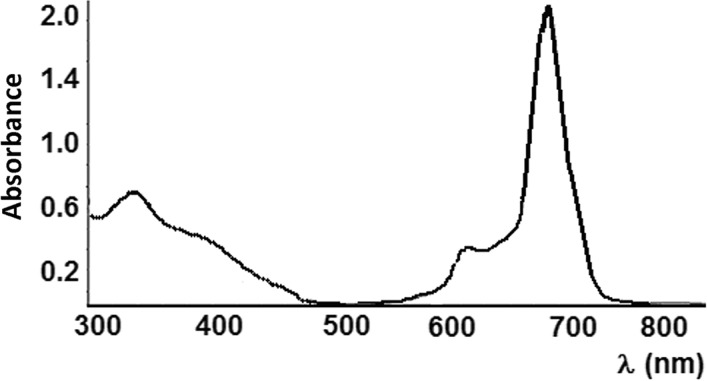
Absorption spectra of 1.38 × 10^−4^ M In-Pc in DMSO

The absorption spectrum of In-Pc in DMSO solution was recorded. B-band (Soret) appears in ultraviolet light area (300–400 nm) - around 345 nm, and a strong absorption in Q-band (Q-band splitting) appears in visible and near-infra-red light region (600–800 nm) - at 635 nm and 685 nm. The studied complex was tested to act upon the Beer's law for concentrations ranging from 0.5 to 7 × 10^−6^ mol/L In-Pc (Figure 1).

The non-aggregation status of In-Pc was confirmed by the absorption spectra with a single Q band with high molar extinction coefficient (8.5 × 10^4^ M^−1^.cm^−1^). Chlorine atom on axial position prevents dimerization/aggregation by restraining the overlap of two/many molecules in solution.

### Singlet oxygen generation

**Figure 2:**
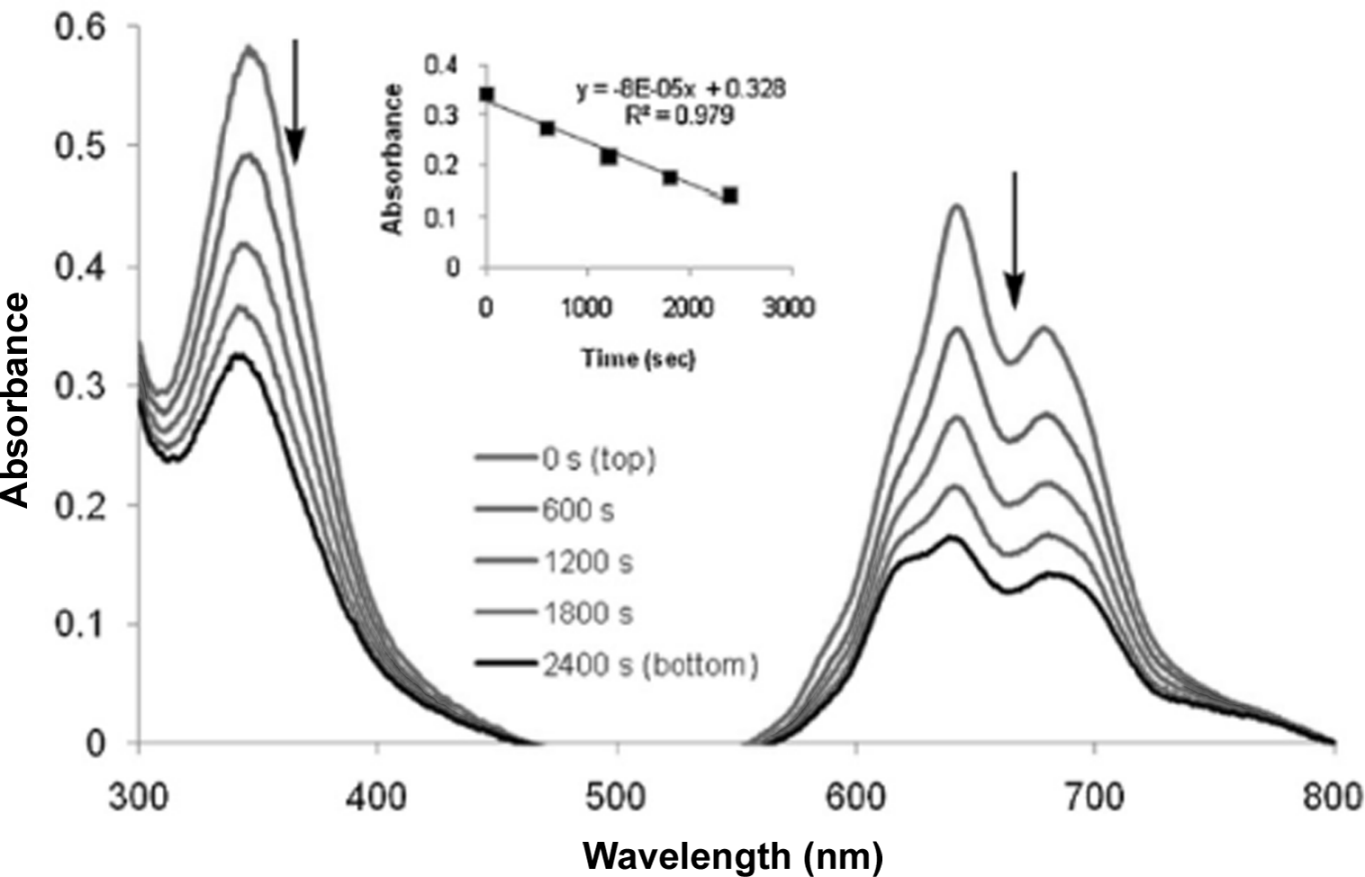
DPBF photo-oxidation due to the singlet oxygen formation of In-Pc

We have investigated In-Pc for the singlet oxygen generation as the main reactive oxygen species inducing damage on cell components. The complexes with non-transition metal such as indium (III) are still insufficiently studied. We have used the DPBF photo-oxidation method to investigate the singlet oxygen formation (Figure 2) and to register it as singlet oxygen quantum yield (ΦΔ).For proving that In-Pc loaded in tumor cell that are subjected to irradiation is generating intracellular singlet oxygen, we have quantified this species in the *in vitro* cellular model (Table 1). Hence, when SH-SY5Y cells were loaded with In-Pc at 10 μg/mL and then irradiated, singlet oxygen was generated almost at the same level (0.533) with singlet oxygen generated in non-cellular model (0.603). When cells were treated with 5 mM sodium azide, a specific radical scavenger, we have obtained a reduction to 23% of the singlet oxygen generation (0.125), proving that, inside the cells the main generated oxygen species is singlet oxygen. As shown in previously published literature [20] singlet oxygen predominates for most photosensitizers. We have also shown that singlet oxygen is the primary reactive oxygen species generated by the excitation of photodynamic agents such as our In-Pc in *in vitro* experimental PDT.

**Table 1:** Singlet oxygen generation in SH-SY5Y neuroblastoma cell line loaded with In-Pc and subjected to experimental PDT

Tested system	ΦΔ
Non-cellular In-Pc in DMSO	0.605
Cells loaded with 10 μg/mL In-Pc and irradiated	0.533
Cells loaded with 10 μg/mL In-Pc, irradiated and treated with NaN_3_	0.125

### Toxicological profile of In-Pc

Knowing that each cell type has particular behavior we have established in our system the best *in vitro* In-Pc concentration and incubation time for this particular cell line. We have assessed dark toxicity taking into account the release of lactate dehydrogenase (LDH), the proliferation capacity quantified both as end-point MTS reduction test and as dynamic impedance measurement. The entire procedure work flow was previously published by us in several approaches for various photosensitizers [21–23].

**Figure 3:**
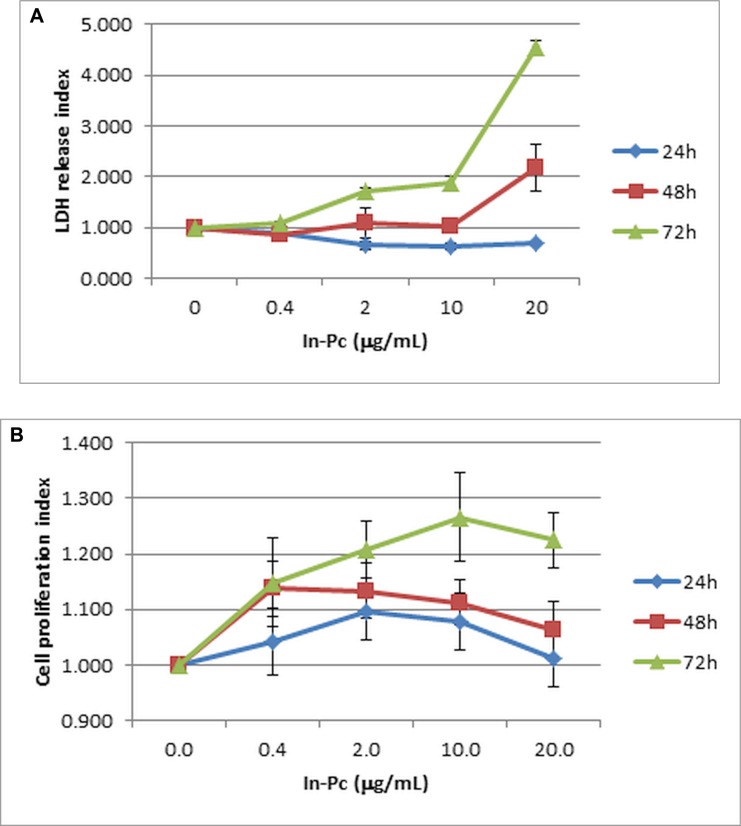
Viability and proliferation of SH-SY5Y cell in presence of In-Pc (**A**) Viability of cell lines in presence of 0–20 μg/mL In-Pc (LDH quantification test from SH-SY5Y cell culture's supernatants expressed as index of LDH release) ***p*** < 0.05 starting with 2 μg/mL; (**B**) Proliferation capacity of cell lines in presence of In-Pc in the range 0–20 μg/mL (MTS quantification test for SH-SY5Y cell culture's supernatants presented as proliferation index); *p* < 0.05 at higher than 2 μg/mL concentration and only after 72 h of incubation.

In our system, using neuroblastoma cell line, we have established that In-Pc starts to significantly hinder cell membrane only after prolonged incubation (after 24 h) and only at higher than 10 μg/mL concentrations (Figure 3A).

The assessment of neuroblastoma cells proliferation capacity shows that, at low In-Pc concentrations there are no statistically significant differences compared to control cells. We have registered a proliferation enhancement for cells incubated for 72 h at > 10 μg/mL In-Pc concentrations (Figure 3B). This result, as surprisingly it seems, was prior reported for MePc in other cellular systems [24] and we do not rule out that, at higher concentrations and longer incubation period, there are epi/genetic alteration that drive the process toward cellular proliferation. These are additional reasons to have the compound loading only up to 24 h.

**Figure 4:**
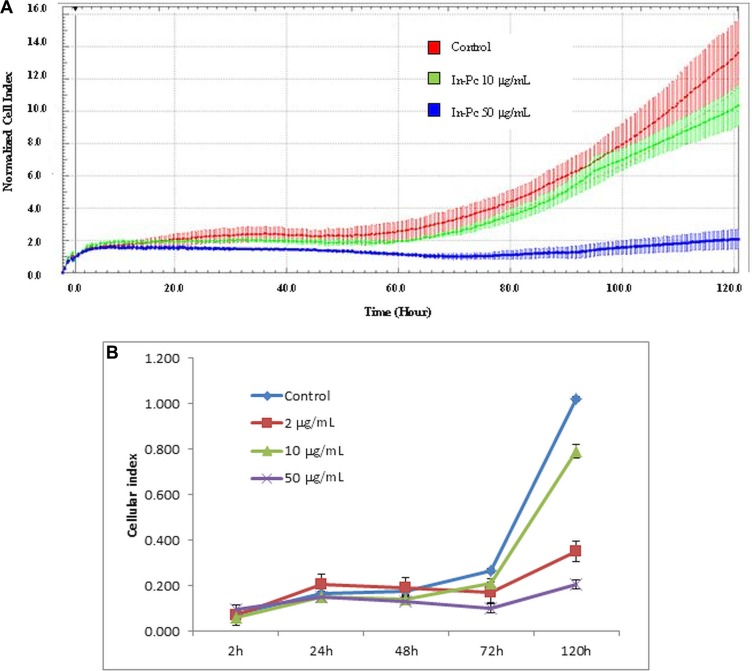
Impedance measurement of SH-SY5Y cell in presence of In-Pc (**A**) 5 days continuous registration of 2,000 cells/well control cells (red), 10 μg/mL In-Pc (green); 50 μg/mL In-Pc (blue); representation of cell index normalized to 2 h as mean ± SD representation of each cell index; *p* < 0.05 between control/10 μg/mL concentration and 50 μg/mL concentration. (**B**) Cellular index for impedance measurements represented as automated CI for 2, 24, 48, 72 and 120 h of SH-SY5Y cultivation (*p* < 0.0001 comparing CI for 120 h cultivation for all tested concentrations).

In order to validate the proliferation results and to strengthen the dark toxicity assessment we have used impedance measurement of cells incubated for 5 days in the tested concentration range (Figure 4A, 4B). The automated cell index (CI) shows a very interesting pattern developed by the cultivated neurons in presence of In-Pc (Figure 4B). Only after 72 h cultivation, the cell line displays different CI in the tested concentration range. 10 μg/mL concentration, further to be used in the experimental PDT, matches the control CI. An interesting situation was found at 2 μg/mL where this low In-Pc concentration hinders cell's proliferation as measured by impedance assay.

### *In vitro* experimental PDT

**Figure 5:**
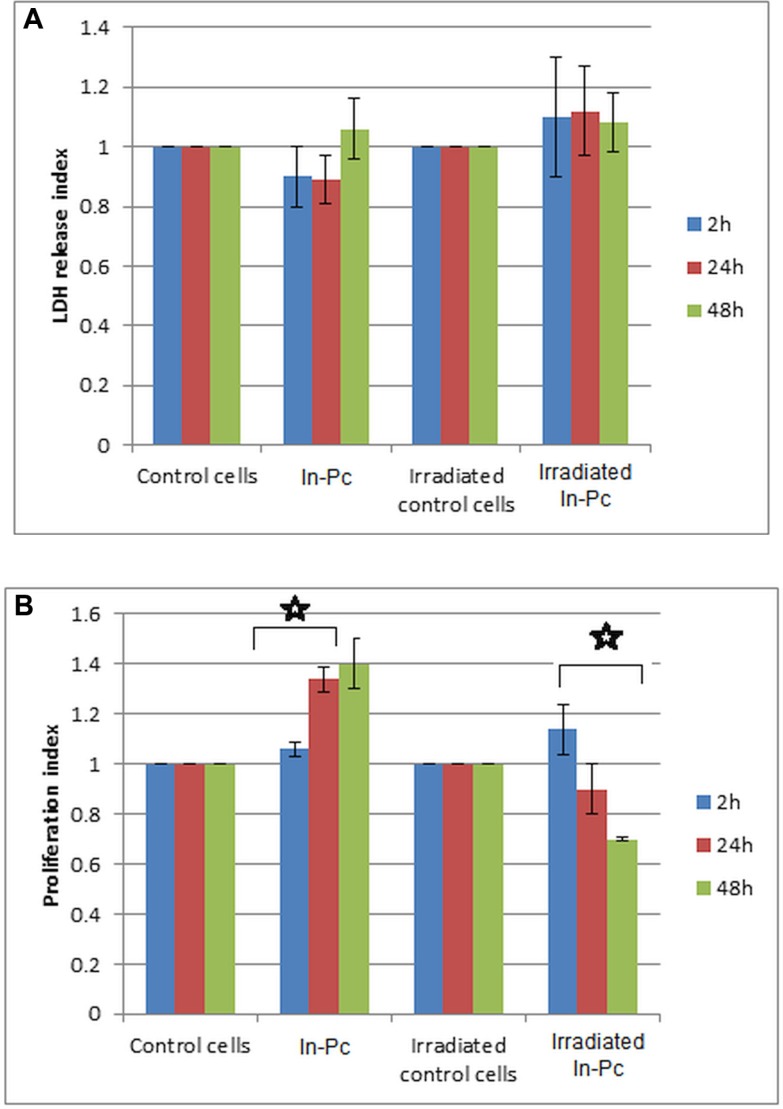
Viability and proliferation of SH-SY5Y cell in presence of 10 μg/mL In-Pc and after experimental PDT (Hg lamp 375 nm with an interferential filter = 680 nm, at a distance of 30 cm; average irradiance 7 × 10^3^ J/m^2^.s) (**A**) LDH release upon experimental PDT; (**B**) proliferation index upon experimental PDT; *p* < 0.005 for irradiated cells after 24 and 48 h post-irradiation.

Neuroblastoma cell line loaded with 10 μg/mL non-toxic concentration of In-Pc was subjected to experimental PDT as described in Material and Methods section. After irradiation, around 50% cells were still alive and the living cells were immediately cultivated for another 72 h. When investigating LDH release, we have registered a slight, non-statistically different increase of the release compared to control cells (Figure 5A). Although LDH release upon irradiation is not different, the registered proliferation decreases constantly upon cultivation (Figure 5B). Thus, after 48 h cultivation, the proliferation index has a mean of 0.6 compared to controls, this value being statistically significant compared to the proliferation of control cells (Figure 5B).

**Figure 6:**
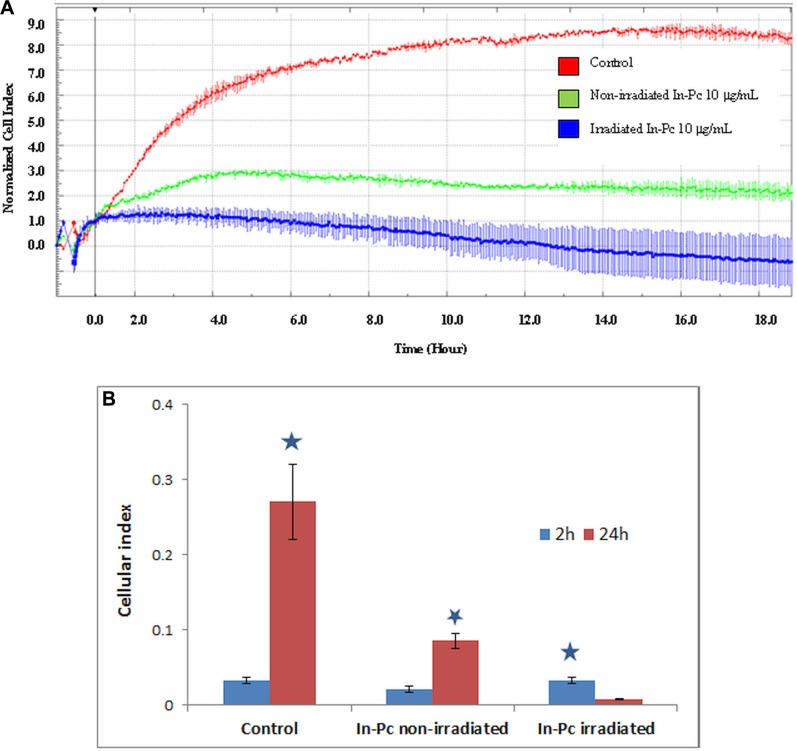
Impedance measurement of SH-SY5Y cell loaded with 10 μg/mL In-Pc and after experimental PDT (Hg lamp 375 nm with an interferential filter = 680 nm, at a distance of 30 cm; average irradiance 7 × **10^3^ J/m^2^.s).** (**A**) Impedance measurement during 24 h post-irradiation of SH-SY5Y cells loaded with 10 μg/mL In-Pc. CI normalized at 1h post seeding in xCelligence E16 plates; (**B**) automated cellular index of irradiated cells loaded with 10 μg/mL In-Pc and subjected to experimental PDT after 24 h of cultivation.

When assessing the impedance measurement (Figure 6) as confirmation of the proliferative capacity of neuroblastoma cells subjected to experimental PDT, we have obtained a marked decrease of proliferation after 24 h of cultivation post-therapy. Automated CI obtained post-irradiation shows a 20 fold decrease in the cellular index for cells loaded with In-Pc after 24 h post-irradiation. This decrease is so marked that it can be registered immediately upon re-cultivation and, after 24 h post-irradiation there were almost no cells that would continue to spread. As expected, we have registered also a 3 fold decrease of CI for non-irradiated but loaded cells. This last ascertain shows that even cells that “escape” the irradiation protocol, although alive (assessed by classical dye exclusion test) have a hindered proliferative capacity.

**Figure 7:**
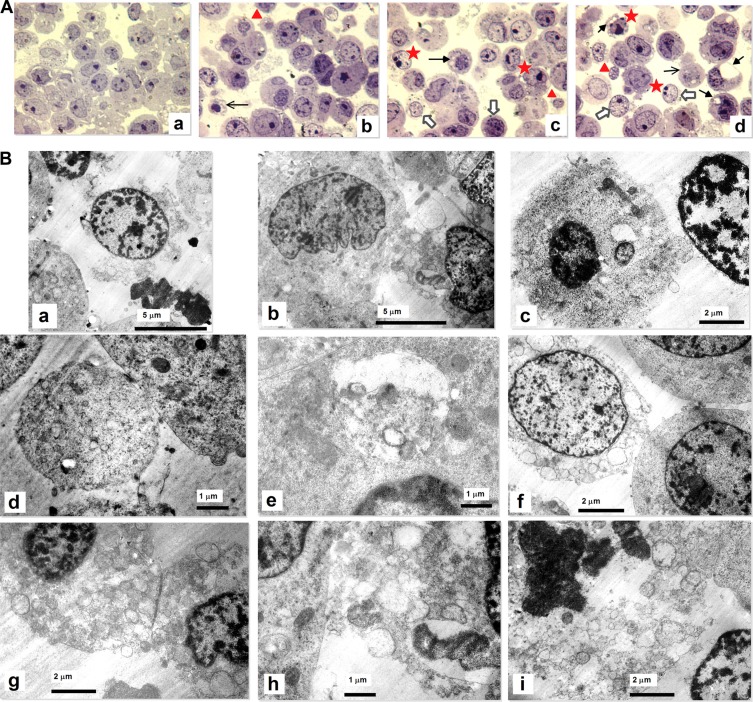
Imagistics of neuroblastoma SH-SY5Y cell line subjected to experimental PDT with 10 μg/mL In-Pc (Hg lamp 375 nm with an interferential filter = 680 nm, at a distance of 30 cm; average irradiance 7 × 10^3^ J/m^2^.s) (**A**) Semi-thin sections. Control cells (a). The most of cells have a normal shaped nucleus with 1-2 nucleoli, surrounded by cytoplasm. Irradiated cells (b, c, d). The presence of apoptosis: cells with vacuoles (compact arrows), nuclei with indentations, abnormal distribution of chromatin at the periphery of nucleus (stars), late phases of apoptotic cells (arrows), apoptotic bodies (triangles), shrinkage of cytoplasm (white arrows). (Light microscopy, 60 × magnification with immersion.) (**B**) TEM images: apoptosis and necrosis are present in irradiated cells. The observed apoptotic changes (a-f) are: clusters of chromatin especially near the nuclear membrane (a, c, f), identated nuclei (b), plasma membrane blebbing (c), shrinkage (a, f), mitochondria with abnormal cristae (h), autophagic vacuoles (e), picnotic nuclei (c), absent nucleoli, apoptotic bodies (d). Necrosis (g-i) is present by cytoplasmic vacuolation, swollen mitochondria and generally disturbed organization, loss of plasma and nuclear membrane integrity, chromatin condensation.

Finding out that cells have a reduced proliferative capacity we have investigated by light microscopy and transmission electron microscopy (TEM) subtle intracellular changes that can explain the obtained parameters (Figure 7). Thus semi-thin sections (700 nm thickness) of epon-embedded cells were stained with toluidine blue (Figure 7A). In contrast to control cells (Figure 7Aa) (normal shaped nucleus, often with 1–2 nucleoli, normal cytoplasm), in the irradiated cells (Figure 7A b, c, d) presence of apoptosis was noticed: cells with many vacuoles, nuclei with indentations, abnormal distribution of chromatin at the periphery of nucleus, some cells with fragmentation the nucleus, shrinkage of cytoplasm, some apoptotic bodies and apoptotic cells in the late phase of apoptosis.

Ultrathin sections (70 nm thickness) of control cells showed nuclei with nucleoli, normal mitochondria and plasmatic membrane without interruptions (not shown). TEM of irradiated cells showed different signs of apoptosis (Figure 7B), starting from early stages - including clusters of chromatin especially near the nuclear membrane, plasma membrane blebbing - to late-stages, with shrinkage, mitochondria with abnormal cristae, autophagic vacuoles, picnotic nuclei, absent nucleoli, apoptotic bodies. Some necrotic cells are also observed presenting cytoplasmic vacuolation, swollen mitochondria and generally disturbed organization, loss of plasma membrane integrity, chromatin condensation. The main cell changes observed by TEM in irradiated cells were apoptotic. Only few images of necrosis were identified. This means that irradiation dose was adequated, the balance between the two processes of cell death being in the favor of apoptosis.

## DISCUSSION

Photodynamic action in biological systems has some clear outlines. The accumulation degree is enhanced in tumor cells due to physiological differences in the neoplastic tissue compared to healthy one [25, 26]. Accumulation of the photosensitizer in the tumor cell depends both on the tumor type and on the photosensitizer properties, this accumulation taking place from 3 to 96 h. Then PDT can be performed with irradiation at a wavelength specific to the photosensitizer. Irradiation produces ROS, mainly singlet oxygen, actively destroying the tumor by inducing apoptosis or necrosis [27].

The most frequently used cell line matching neuroblastoma tumor is SH-SY5Y. The majority of PDT published studies regarding neuroblastoma cell line are concentrated on 5-aminolevulinic acid (5-ALA) as a photosensitizer [28]. This PS is actively engulfed in the first 6–8 h and then more slowly until 12 h [29]. Neuroblastoma cells are sensitive to 5-ALA PDT destruction in comparison to other cell lines [30]. On the contrast, other recent studies have shown that in comparison to other cell lines, carboxylated porphyrins are accumulating in a lesser extent in SH-SY5Y [28] which are less sensitive to ALA/MAL-PDT [31]. Balancing through these reports we have embarked in the study of In-Pc as possible future PDT drug in neuroblastoma.

Studies regarding the effect of central metal atom on the photodynamic activity of phthalocyanine dyes go back more than 25 years ago. MePcs display quantum yields for O_2_^−^ generation ranging from 10^−5^ (Zn-phthalocyanine) to 4.2 × 10^−4^ (Ga-phthalocyanine) [32]. Moreover it was shown that the efficiency of generating O_2_^−^ was apparently uncorrelated with the dyes' phototoxicity [32]. In our system we have shown that singlet oxygen was generated almost at the same level with singlet oxygen generated in non-cellular model, showing that singlet oxygen was the primary reactive oxygen species generated through *in vitro* experimental In-Pc-PDT.

Data regarding PDT usage of In-Pc does not abound. Thus, there is only a recent study reporting an efficient *in vitro* killing (> 60%) of A549 lung cells by In-Pc PDT [33]. In our neuroblastoma model, In-Pc actively destroyed 50% of the cells and hindered the proliferation of the remaining ones. This observation relies probably on more profound intra-cellular mechanisms that are deeply affected upon irradiation. The effect can be due to the well-known glioblastoma characteristic, namely the constitutive activation of the NF-κB, key regulator of various physiological processes such as cell proliferation or apoptosis. As data regarding PDT with In-Pc for glioblastoma are still missing, we can only extrapolate that NF-kB modulation could improve the efficacy of In-Pc-PDT as it was already published for ALA-PDT [34].

Cells subjected to PDT could undergo apoptosis or necrosis depending on the applied light dose, as in general a low light dose can induce a massive apoptosis while a high dose of light preferentially leads to massive necrotic effects [35]. As it is known that singlet oxygen targets among other biomolecules, nucleic acid bases [20], we can ascertain that the apoptotic mechanisms that were highlighted by us reside mainly on singlet oxygen generation upon experimental PDT. We always aim to induce apoptosis in our experimental approaches as apoptosis generates no deleterious side effects compared with necrosis which is mostly seen as a disadvantageous mechanism. Necrosis can lead to an uncontrolled extracellular release of biomolecules inducing an inflammatory milieu in the nearby tissue [36]. Moreover, apoptosis is important in inducing an immunogenic character of tumor cell as PDT leads to activation of an antitumor immune response by which some tumor cells are destroyed *via* cytotoxic T cells [33]. As a consequence, it is of great importance to know the underlying action mechanism of a new drug upon tumor cells killing in order to improve a certain therapeutic protocol [37, 38–40]. By our PDT experimental procedure we could induce mainly apoptosis in SH-SY5Y cells. In-Pc induced in neuroblastoma cell line extensive apoptotic damage as investigated by TEM, as recently proven with other MePc in HeLa cells [41]. Another interesting issue in NB tumor is that it has a complex form of differentiation. Thus advanced cellular differentiation directly influences the susceptibility of NB to PDT. Hence hematoporphyrin uptake by undifferentiated SH-SY5Y cell line is lower by comparison with differentiated cells, but more susceptible to PDT-induced oxidative stress killing. Undifferentiated cells are subjected to cycle arrest at G2/M phase, mitochondrial apoptotic pathway activation, and sustained phosphorylation of Akt/GSK-3β and ERK when subjected to ROS-generated by PDT [42]. The fact that one can modulate using PDT intracellular signalling that can enhance apoptotic pathways like in other brain tumor [43, 44] seems a good, clinically-friendly future approach.

Furthermore, in the flow of preclinical research, establishing an animal model for experimental PDT in NB would be the next step for In-Pc as future therapeutic agent. Hence, the chorioallantoic membrane of fertilized chicken eggs is a simple model for studying PDT-induced effects using *in vivo* microscopy [45]. Advancing in animal models one can imagine that mouse strains (e.g. BALB/c or C57BL/6), are commonly used for *in vivo* models for PDT in melanoma, fibrosarcoma, lymphoma, or malignant glioma [46]. The PS concentration to be used in *in vivo* models should be tailored according to animal used, for instance in a study regarding PDT with ALA in rat glioma model (BT4C multicellular tumor spheroids) ALA was intra-peritoneally delivered in a dose of 125 mg/kg. Thus, the PS dose is to be tailored depending on animal model and should be correlated with other parameters essential for PDT efficacy, such as the rate of light delivery [47]. Also the nature of solvent used for PS administration is another factor to be considered, as the major downside of many potential PS is their low solubility in water and respectively in biological fluids [48]. An ideal delivering formula for PS should guarantee a stable dispersion of PS in aqueous systems, a lowermost quantity of adjuvants while therapeutic dosage of PS at tumor site remaining unaffected. By tradition, DMSO is preferred for obtaining PS soluble and stable in water and biological media [49]. Our In-Pc formula for *in vitro* assessment of SH-SY5Y was prepared taking into account the nature of PS, a stock solution in DMSO was prepared and further working solution in serial dilution in cell culture media was obtained, hence the final DMSO concentration in the tested In-Pc range doses was under 1% (v/v), avoiding a toxicity which DMSO could exhibit in certain experimental conditions according to other published reports [50].

The further thorough investigations regarding In-Pc in cancer treatment are necessary as data concerning this type of PS in cancer therapeutics area do not abound as we already pointed out. Such fine explorations could be expanded both at biological and chemical level, this avenue being a tempting and pristine new one assessment in PDT field. Thus, at biological level our study discloses new possibilities for testing its efficacy in other cancers types such as malignant melanoma [51] or breast cancer [52], generally accessible tumors for delivering a particular PS in a targeted manner. In addition, the chemistry approaches of In-Pc prevails over its biological assessments and reach new levels by exploring for instance PEGylated poly(d,l-lactide-co-glycolide) nanoparticles loaded with In-Pc in an attempt to increase the PDT efficacy using MCF7 breast cancer cell line [52]. Coupling In-Pc with antibodies could be another approach, a still insufficiently explored path in the quest for improving targeted delivery. Hence binding PS to specific tumor - antigens generates “photo-immunoconjugates” that could become nowadays an attractive strategy in PDT [53]. Studies could be expanded beyond cellular membrane as interaction of various Pc with DNA structures become an actual endeavor that could be extended also to metallated Pcs [54].

## MATERIALS AND METHODS

### Photosensitizer

Chlorine Indium (III) phthalocyanine (Aldrich Chemicals, USA) was purified by the sublimation technique using nitrogen gas as the carrier, and characterized by specific techniques as follows:*^1^H NMR (d, Hz):* 9.65 and 8.47 (8H). *FTIR (KBr) (cm^−1^*): 2950, 1716, 1631, 1557, 1458, 1261, 1184, 1096, 1018, 971, 850, 805, 747, 551. *UV–Vis* λ_max_ *(nm):* 615, 561, 428 (in NMP). *MS:* (m/z) (FAB^+^) 951. *Elemental analysis* for C_60_H_60_N_4_InCl, Calc.: C, 72.98; H, 6.12; N, 5.67. Found: C, 72.85; H, 6.03; N, 5.72%.

^1^*H NMR* spectra were recorded at Bruker Avance-III spectrometer with 600.13 MHz frequency. Spectra were acquired at T = 298 K, in CDCl3. Chemical shifts were measured relatively to internal standard (tetramethylsilane, δ = 0.00 ppm).

*UV-vis absorption spectra* were recorded in 250– 900 nm spectral regions with SPECORD M400, Carl Zeiss Jena spectrophotometer with double beam and equipped with a microprocessor. Quartz cuvettes with 0.5–2 cm optical path lengths and containing 1 ml of cell suspension each were used. The structure of the tested photosensitizer is presented in the Supplementary Material.

### Singlet oxygen generation

The applied method uses singlet oxygen scavengers or quenchers. 1,3-diphenylisobenzofuran (DPBF) is known to be an exclusive and standard quencher in organic solvents. As soon as singlet oxygen is generated, it can be trapped in this singlet oxygen quencher. The disappearance of the quencher can be spectroscopically determined in air (no oxygen bubbled) using the relative method, reported by literature [55]. For all our measurements ZnPc has been used as reference [56] and DPBF as chemical quencher [57]:

$$\begin{array}{lll} \Phi_{\Delta } & = & \Phi_{\Delta }^{\text{Std}}\cdot \frac{R_{\text{DPBF}}I_{\text{abs}}^{\text{Std}}}{R_{\text{DPBF}}^{\text{Std}}I_{\text{abs}}}\\ I_{\text{abs}} & = & \frac{\alpha \text{SI}}{N_{A}} \end{array}$$

where:

Φ_Δ_^Std^is the singlet oxygen quantum is yield for the standard, e.g. ZnPc; R _DPBF_and *R*_DPBF_^Std^are the DPBF photodegradation rates in the presence of a sensitizer under investigation and the standard respectively; I_abs_ and I^Std^_abs_ are the rates of light absorption by the sensitizer and standard, respectively; ΔA, Dt, V and ε are change in absorbance, in irradiation time, volume and molar absorption coefficient; A is the fraction of absorbed light; I is the light intensity; S is the irradiated cell area (cm^2^); N_A_ is Avogadro's constant (mol^−1^).

As a control for singlet oxygen intracellular generation upon photoactivation, SH-SY5Y cells were treated with sodium azide (NaN_3_) 5 mM prior to irradiation. 5 mM NaN_3_ concentration is lower than the toxic level (10 mM) [58], and all the solutions have been used after only 1–1.5 h after preparation, in order to avoid azide toxicity [59].

### Cell line and maintenance

SH-SY5Y, human neuroblastoma cell line, was obtained and authenticated from ECACC (ECACC catalogue no. 86012802). Cultivation was performed using 1:1 mixture of ATCC-formulated Eagle's Minimum Essential Medium (Catalog No. 30–2003), and F12 Medium, added with 10% foetal bovine serum (FBS), 1% penicillin and 1% streptomycin (Invitrogen, CA). The cells were seeded in low density, maximum 1 × 10^4^/cm^2^ and kept in air (95%), carbon dioxide (CO_2_) 5% at 37°C. Cells were counted, viability registered and re-seeded in new flasks for further cultivation when reaching 80–90% confluence. Cell counting and live cell registration was done throughout cultivation and experimental procedures described herein using an automated counter - Countess II Automated Cell Counter (Thermo Fisher Scientific).

### Experimental irradiation

Cell cultures were incubated in In-Pc for 24 h in the dark at 37^°^C and afterward washed 2 times in culture medium for removal of free photosensitizer. Trypsinized cell cultures were resuspended at 3 × 10^5^ cells/mL concentration in cell culture medium. Cell suspensions were irradiated with an Hg medium pressure lamp (375 W), Romlux, Romania. A 600 nm glass cut off filter (Schott) and a water filter were used to filter off ultraviolet and infrared radiations respectively. Illumination was carried out on 20 mL samples in a Petri dish (100 × 15 mm) using a medium pressure Hg lamp (375 nm) with an interferential filter = 680 nm, at a distance of 30 cm (average irradiance 7 × 10^3^ J/m^2^.s). An interference filter (Intor, 680 nm with a band width of 40 nm) was additionally placed in the light path before the sample. Light intensities were measured with a power meter with detector incorporated (ICPE). During irradiation, the cell suspension was constantly stirred. After irradiation, cells were washed 2 times in culture medium for removal of cell debris generated during irradiation and subjected to further testing.

### Evaluation of cellular parameters in experimental PDT

*Cell membrane integrity* was quantified using extracellular lactate dehydrogenase (LDH) release with standard Cytotox96 Non-Radioactive Cytotoxicity Assay kit (Promega Corporation). The level of LDH released in cellular supernatant was determined by measuring the optical density recorded at 490 nm (OD 490 nm).

*Cell metabolism* was evaluated using standard CellTiter 96AQueous One Solution Cell Proliferation kit (Promega Corporation) that is based on MTS as a tetrazolium compound [3-(4,5-dimethylthiazol-2-yl)-5-(3-carboxymethoxyphenyl)-2-(4-sulfophenyl)-2H-tetrazolium] and an electron coupling reagent (phenazineethosulfate - PES). PES has enhanced chemical stability, which allows it to be combined with MTS to form a stable solution. The quantity of formazan product as measured by the amount of 490 nm absorbance is directly proportional to the number of living cells in culture. The absorbance of each sample was recorded with an ELISA plate reader. Results are expressed as optical density recorded at 490 nm (OD 490 nm).

*Real-time monitoring of cell response using impedance technology* was performed on collagen I (Sigma C7661) 5 μg/mL in 0.1 M NaHCO_3_ coated E-16 plates (Roche, Penzberg Upper Bavaria, Germany, catalogue no.05469830001), compatible with RCTA-DP system (Roche Applied Science). Using our already published general impedance protocol [60, 61] 2,000 neuroblastoma cells were plated in each well and readings were collected at 1 minute intervals for 72 hours and the results reported as normalized Cell Index (CI) to the time just before compound addition. The assay system expresses impedance in arbitrary CI units. Each situation was quadruplicated. When cell treatments were performed, normal cell media was replaced with media containing the phthalocyanine at each indicated concentration.

### Statistics

3 separate experiments were performed and samples were used in triplicates/quadruplicates. The presented results are the mean ±SD of these 3 experiments. For proliferation (MTS test) or LDH release tests cellular index was calculated by dividing individual OD for actual samples to each appropriate cell controls. For impedance measurement each sample was quadruplicated and xCELLigence Real-Time Cell Analyzer Software that is incorporated in the RCTA-DP system, automatically calculates mean ±SD for each sample. Statistical analysis was carried out with SPSS software, using OneWayAnova test of variance followed by post-hoc Bonferroni correction. Statistical significance was reached for *p* value < 0.01.

### Transmission electron microscopy (TEM)

For conventional TEM, control and subjected to PDT, neuroblastoma cells were pelleted down and immediately fixed with a fresh solution of 2.5% glutaraldehyde/paraformaldehyde in phosphate buffer saline (PBS), 2 hours at room temperature. After washing in PBS the cell pellets were post-fixed in 1% osmium tetroxide in Sorensen buffer, 1 h at room temperature. Embedding was done in Epon812 after the dehydration in successive concentration series of ethanol (50, 70, 90 100%) [62]. Semi-thin sections (700 nm) were stained with toluidine blue 1% and examined under a light microscope (Nikon TS 100 Eclipse Inverted Microscope) with a 60× oil immersion objective. Ultrathin sections were cut at 70 nm with a Leica EM UC7 ultramicrotome and were stained with uranyl acetate and lead citrate and examined with a transmission electron microscope (FEI Tecnai F20).

## CONCLUSIONS

In contrast to radiation therapy, PDT is a non-ionizing radiation that can be applied repeatedly without the known cumulative long-term complications. The possibility to use diamagnetic MePc for PDT as therapy of invasive brain tumor adds new players in the therapeutical approach of this disease. We have tested In-Pc as future PDT agent to be developed in brain tumor therapy. This PS displays good toxicological behavior and upon specific light activation, it actively triggers singlet oxygen inducing massive and long-term apoptosis in tumor cells. As it has a chemically maneuverable structure, future to be developed direction can be the combination of nanoparticles with diamagnetic MePc [63] in order to enhance penetrability in tumor that reside upon brain blood barrier. This possible new drug relies is the fact that there are still no efficient therapy for children's NB, and as chemo-resistant tumor cells develop, this new agent can target a particular cell subpopulation. Acknowledging that in neuroblastoma, inhibitors of pro-tumoral signalling pathways [64, 65] or specific anti-tumoral receptor activators [66] or drugs that trigger angiogenesis [67] can add to the therapeutical armentarium, we can imagine future agents that have complex biological behavior [68]. As such, for the future therapy a triad comprising a carrier nanoparticle, the photosensitizer and a compound interacting with specific receptors/specific vasculogenesis markers/intracellular signalling pathways can hinder, taken as a whole, the complex pro-malignant processes.

## SUPPLEMENTARY MATERIALS


