# Supplementary material for: Toxicological and efficacy assessment of post-transition metal (Indium) phthalocyanine for photodynamic therapy in neuroblastoma
Source: Oncotarget. 2016 Sep 10;7(43):69718–32. doi: 10.18632/oncotarget.11942 (PMC5342510; doi:10.18632/oncotarget.11942)
Supplement: Supplementary file 1 [file oncotarget-07-69718-s001.pdf]

Toxicological and efficacy assessment of post-transition metal (Indium) phthalocyanine for photodynamic therapy in neuroblastoma

Supplementary Materials

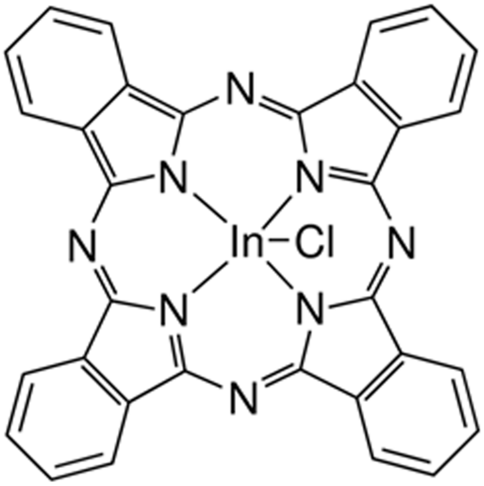

Supplementary Figure S1: The structure of Chloride Indium (III) phthalocyanine.
